# The Organizational Halo: How Perceived Philanthropy Awareness Curbs Abusive Supervision via Moral Pride

**DOI:** 10.3390/bs15121706

**Published:** 2025-12-09

**Authors:** Dong Ju, Yan Tang, Shu Geng, Ruobing Lu, Weifeng Wang

**Affiliations:** 1 Business School, Beijing Normal University, Beijing 100875, China; dongju@bnu.edu.cn (D.J.); tangyan@mail.bnu.edu.cn (Y.T.); wangweifeng@mail.bnu.edu.cn (W.W.); 2 Peking University Education Foundation, Peking University, Beijing 100871, China

**Keywords:** abusive supervision, affective events theory, moral pride, corporate philanthropy, moral reputation

## Abstract

Abusive supervision remains a pervasive and damaging phenomenon in organizations, prompting a critical need to understand preventive mechanisms. We adopt a leader-centric, actor-focused perspective to investigate how a positive organizational context can inhibit leaders’ destructive behaviors. Drawing on Affective Events Theory (AET), we propose that leaders’ awareness of their organization’s philanthropic activities serves as a positive, morally salient event that generates feelings of moral pride. This pride, in turn, is theorized to reduce the likelihood of abusive supervision. Furthermore, we posit that this process is contingent on leaders’ moral reputation maintenance concerns, such that the negative relationship between moral pride and abusive supervision is stronger for leaders who are highly concerned with being perceived as moral. We tested this model using a three-wave survey study involving 434 leaders. The results support our hypotheses, indicating that perceived philanthropy awareness is positively associated with moral pride, which, in turn, predicts lower abusive supervision. This indirect effect is significantly stronger for leaders with high moral reputation maintenance concerns. Our findings contribute to the literature by identifying a novel, positive, and self-regulatory pathway for preventing abusive supervision and showing that applying AET to understand how macro-level organizational good deeds can translate into improved micro-level leader conduct.

## 1. Introduction

Abusive supervision, defined as “subordinates’ perceptions of the extent to which supervisors engage in the sustained display of hostile verbal and nonverbal behaviors, excluding physical contact” (Tepper, 2000, p. 178), is a destructive force in the modern workplace. It undermines organizational functioning by inflicting psychological distress on employees, fostering team-level conflict and resentment, and ultimately leading to detrimental behaviors such as decreased performance and increased deviance against the organization (Farh & Chen, 2014; Mackey et al., 2017; Wang et al., 2023). While an extensive body of research has thoroughly documented the detrimental consequences of abusive supervision on subordinates, this prevailing focus on downstream harm offers an incomplete picture. A smaller, emerging stream of inquiry has begun to explore subordinate-driven strategies to curtail such behavior (M. Huang et al., 2023), yet we still need a better understanding of how to curb abusive supervision from various perspectives. A potentially more fundamental approach is to move beyond documenting the damage or exploring recipient reactions and instead identify the upstream, contextual factors that can proactively prevent abusive supervision from emerging. This leaves a critical gap regarding the role of the broader organizational environment in either enabling or inhibiting destructive leadership. Specifically, we lack a clear understanding of how positive, organizational-level variables create a systemic context that proactively inhibits such behavior. Therefore, our study shifts the analytical lens from the consequences of abuse to its proactive mitigation. We specifically investigate how positive, organizational-level variables create a context that can morally fortify leaders, thereby reducing the likelihood of abusive behavior and offering a more systemic solution to this pervasive problem. The research question is “How does leaders’ awareness of their organization’s corporate philanthropy (a positive organizational-level variable) curb abusive supervision, and what is the mediating role of moral pride and the moderating role of moral reputation maintenance concerns in this process?”

We propose that a leader’s awareness of their company’s philanthropic efforts acts as a positive workplace driver. Corporate philanthropy has become an undeniable and growing feature of the global business landscape, with corporate giving reaching over USD 20 billion in 2017 alone and showing a consistent upward trend (Cha & Rajadhyaksha, 2021). To explain the mechanism through which corporate philanthropy awareness translates into positive leader behavior, we draw upon Affective Events Theory (AET; Weiss & Cropanzano, 1996). AET posits that events in the work environment trigger affective reactions in individuals, which in turn shape their attitudes and behaviors. We argue that leaders’ awareness of their organization’s pro-social actions is a morally laden factor that elicits the self-conscious emotion of moral pride. This positive emotion, we contend, serves as a psychological resource that makes leaders less likely to engage in abusive supervision. Furthermore, we propose a crucial boundary condition for this effect: leaders’ moral reputation maintenance concerns. We theorize that the function of moral pride is most potent for leaders who are highly motivated to maintain a moral image, as abusive behavior would directly contradict this valued self-concept.

This study makes several key contributions. First, this research makes a significant contribution to the literature on abusive supervision by identifying and empirically testing a novel, organization-level antecedent—perceived corporate philanthropy awareness. To date, the search for factors that trigger abusive supervision has predominantly focused on leader-centric and dyadic-level variables. A substantial body of research, synthesized in multiple meta-analyses (Mackey et al., 2017; Tepper et al., 2017), has established that abusive behaviors often stem from leaders’ individual characteristics (e.g., neuroticism, low self-control), psychological states (e.g., ego depletion, psychological distress), and perceptions of subordinates (e.g., low performance, interpersonal conflict). While some research has extended this view to include the immediate work environment, it has primarily examined internal organizational factors, such as perceptions of injustice or abusive supervision environment (Hoobler & Hu, 2013; Priesemuth et al., 2014; Tepper, 2007).

Second, our study moves beyond these established antecedents by theorizing that an organization’s external pro-social actions can have a profound, cascading effect on the internal quality of its leadership. By linking an organization’s macro-level ethical posture (its philanthropic engagement) to its leaders’ micro-level interpersonal behavior, we answer calls for a more multilevel understanding of destructive leadership (Mathieu & Chen, 2011). We propose that the ethical fabric of an organization, as demonstrated through its contributions to the wider community, creates a positive, affective context for its leaders, which, in turn, discourages destructive interpersonal conduct. In doing so, we are among the first to empirically bridge the corporate social responsibility (CSR) literature with the abusive supervision literature, offering a new, positive, and context-based avenue for the prevention of abusive supervision.

Third, we contribute to the application of AET by demonstrating how a macro-level organizational factor, as perceived by individual leaders, can trigger a specific moral emotion that influences micro-level interpersonal conduct.

## 2. Theoretical Background and Hypothesis Development

### 2.1. Theoretical Background: Affective Events Theory

Our theoretical framework is grounded in Affective Events Theory (AET; Weiss & Cropanzano, 1996). AET was proposed to address a gap in organizational research, which had traditionally focused on stable job features (e.g., pay, autonomy) and their cognitive influence on static attitudes like overall job satisfaction (Weiss & Cropanzano, 1996). Weiss and Cropanzano (1996) argued that this perspective overlooked the dynamic, moment-to-moment emotional lives of employees, which are critical for understanding both behaviors and attitudes. AET’s core proposition is that the work environment is not just a collection of stable “features” but a stage for unfolding “events” (Weiss & Cropanzano, 1996, p. 11). These events are the proximal causes of employees’ fluctuating affective reactions (i.e., discrete emotions and more diffuse moods), which, in turn, have a powerful influence on their attitudes and behaviors.

Since its introduction, AET has been widely applied to understand how workplace events shape leader and employee behaviors. A significant body of research has applied AET to explain the link between negative workplace events and destructive outcomes. For instance, scholars have explicitly applied AET to leadership, arguing that leaders’ affective states are critical to the leadership process. Negative events, such as leaders’ perceptions of interactional injustice, have been shown to trigger state negative affect (an affective reaction), which, in turn, predicts leaders’ engagement in abusive supervision (Hoobler & Hu, 2013). Similarly, Pan and Lin (2016) argue that a supervisor’s negative affect is a proximal, automatic trigger for aggressive (i.e., abusive) behaviors. This research stream firmly establishes destructive leadership as a form of affect-driven behavior, often precipitated by negative workplace events and the subsequent negative emotions they create.

Concurrently, AET has been applied to understand the outcomes of positive events. Research on corporate social responsibility (CSR) has increasingly adopted an affective lens. For example, scholars have framed CSR initiatives as positive, organization-level events that when perceived by employees, elicit specific positive moral emotions such as gratitude and compassion (Guzzo et al., 2020). Lee et al. (2015) found that philanthropic sponsorship evokes positive emotions (pleasure and arousal), which, in turn, influence brand equity. Schaefer et al. (2024) explicitly combined appraisal theory (a core component of AET) with social identity theory to show how employees’ positive evaluations of CSR lead to the powerful emotion of organizational pride. We build directly on this foundation, conceptualizing perceived corporate philanthropy as a positive, macro-level affective event that influences leaders’ affect, cognitions, and subsequent behavior.

### 2.2. Affective Events Theory and Philanthropy Awareness

Affective Events Theory (AET) provides a framework for understanding how the workplace environment influences employees’ emotions and behaviors (Weiss & Cropanzano, 1996). The core premise of AET is that the workplace is characterized by events that trigger emotional responses (affective reactions), which subsequently influence affect-driven and judgment-driven behaviors. These events are not necessarily dramatic; they can be minor everyday occurrences. We extend this logic by proposing that leaders’ awareness of their organization’s corporate philanthropy constitutes a significant, positive affective event.

Corporate philanthropy involves an organization’s discretionary contributions of resources to the community to improve social welfare (Godfrey, 2005). It can help build brand loyalty, enhance reputation, secure employee commitment, and establish the company as a responsible corporate citizen (Cha & Rajadhyaksha, 2021; Gautier & Pache, 2015). When leaders become aware of these “good deeds,” it serves as a morally salient event. According to AET, individuals cognitively appraise events to determine their emotional responses (Lazarus, 1991). We argue that leaders who perceive their organization as engaging in socially desirable and valued actions are likely to appraise such events positively. This positive appraisal, rooted in the moral domain, is expected to generate a specific self-conscious emotion: moral pride. This pride is generated when one attributes a socially valued outcome to oneself or one’s group (Mascolo & Fischer, 1995). By being part of a moral organization, leaders can experience a vicarious sense of moral accomplishment, leading to feelings of pride. This aligns with a rich body of research demonstrating that CSR initiatives are a powerful source of positive employee emotions, such as organizational pride (Mansour et al., 2023), and enhance employee–company identification, which is often mediated by perceived external prestige (Kim et al., 2010). Therefore, we propose the following hypothesis:

**Hypothesis** **1.** *Perceived philanthropy awareness is positively related to moral pride.*

### 2.3. The Mediating Role of Moral Pride

We further theorize that the moral pride generated from philanthropy awareness serves as the mechanism linking this awareness to a reduction in abusive supervision. This proposition is supported by a growing body of research demonstrating that an organization’s pro-social actions can profoundly shape the internal affective states of its members. For instance, Ng et al. (2019) found that employees’ perceptions of their firm’s corporate social responsibility activities were a significant driver of organizational pride, which in turn fostered positive outcomes like organizational embeddedness. This establishes a strong precedent for our argument’s first stage: when leaders perceive their organization as engaging in morally commendable actions related to philanthropy, it enhances their own sense of pride through their affiliation.

AET posits that affective states directly influence behaviors (Weiss & Cropanzano, 1996). Moral pride is a positive, self-enhancing emotion (Mascolo & Fischer, 1995) that can build psychological resources. According to the broaden-and-build theory of positive emotions (Fredrickson, 2001), positive emotions like pride broaden an individual’s thought–action repertoires and build enduring personal resources. When leaders experience moral pride, their enhanced sense of self-worth and positive affect can serve as a buffer against stressors and frustrations that often trigger abusive outbursts (Mawritz et al., 2017). The predominant self-regulation perspective on abusive supervision suggests that abuse often occurs when leaders’ resources are depleted, leading to a failure of self-control (Barnes et al., 2015). By generating a positive emotional and psychological resource, moral pride could bolster leaders’ self-regulatory capacity, allowing them to be more capable of managing their impulses and responding constructively to subordinate-related challenges. A leader who feels morally proud is in a psychological state that is incompatible with the hostile and demeaning actions that constitute abusive supervision (Michie, 2009; Sanders et al., 2018). Furthermore, research by Sanders et al. (2018) established that pride positively predicts leaders’ ethical behavior. Thus, we propose that moral pride mediates the relationship between philanthropy awareness and abusive behavior.

**Hypothesis** **2.** *Moral pride mediates the relationship between perceived philanthropy awareness and abusive supervision.*

### 2.4. The Moderating Role of Moral Reputation Maintenance Concerns

Affective Events Theory suggests that dispositional factors can shape the link between emotional states and subsequent behaviors (Weiss & Cropanzano, 1996). In our model, we argue that moral pride constitutes a psychological resource to refrain from abusive behavior. However, the motivation to activate this resource is not uniform. We posit that leaders’ moral reputation maintenance concerns (MRMCs) represent this critical motivational contingency.

We conceptualize MRMCs as a stable individual difference reflecting an “other-focused motivation” centered on preserving the social perceptions of one’s morality (Baer et al., 2015; Cavazza et al., 2015). While all leaders may feel pride, only those highly concerned with being seen as moral will be strongly motivated to ensure that their actions (i.e., not being abusive) align with that positive affective state. We propose that leaders’ moral reputation maintenance concerns (MRMCs) are a critical individual difference that governs the strength of moral pride’s self-regulatory function. MRMCs represent a domain-specific form of general reputation maintenance concerns that reflect the “psychological pressure to preserve others’ perceptions of one’s morality” (L. Huang et al., 2025). They capture an “other-focused motivation” within one’s moral self-regulation, which is centered on managing the social perceptions of one’s character (Cavazza et al., 2015; Vonasch et al., 2018).

Individuals with high MRMCs are highly attuned to their social environment and are strongly motivated to maintain a favorable moral image in the eyes of others (Leary & Kowalski, 1990). A positive moral reputation is a valuable social asset that can confer status, trust, and influence, and individuals will often incur significant costs to protect it (Vonasch et al., 2018). For leaders, who typically operate under heightened scrutiny, managing their reputation is a crucial aspect of their role. Therefore, leaders with high MRMCs are particularly sensitive to situations that could either bolster or threaten their moral standing and are motivated to engage in impression management to secure a positive image.

We argue that this strong reputational focus creates a powerful contingency for the behavioral consequences of moral pride. When leaders with high MRMCs experience moral pride—an emotion that reinforces their positive moral self-concept—this internal state becomes highly salient. Engaging in abusive supervision, a clear and often visible moral transgression, would create a stark and threatening inconsistency between this salient, positive self-view and their actions. This creates a “hypocrisy gap” that is particularly threatening to those concerned with their public image, as observers are known to judge moral hypocrisy with exceptional harshness (Barden et al., 2005). For leaders with high MRMCs, the potential reputational damage of being perceived as a hypocrite—one who feels morally proud yet acts abusively—is a significant deterrent. Thus, for these leaders, the experience of moral pride activates a potent self-regulatory motivation to align their behaviors with their desired public image, thereby powerfully strengthening the negative relationship between moral pride and abusive supervision.

In contrast, leaders with low MRMCs are, by definition, less concerned with how others perceive their morality. While the moral pride they experience from philanthropy awareness may still provide them with positive affective resources and a general buffer against negative impulses (as proposed in H2), the specific powerful motivation to align their behaviors with a public moral image is absent or weak. The link between feeling morally good and social consequences of acting immorally is less salient for these leaders. Consequently, the inhibiting effect of their engagement in abusive supervision is expected to be substantially weaker. Taken together, we posit that the self-regulatory benefits of moral pride are most fully realized when a leader is also motivated by a strong desire to be seen as a moral person.

**Hypothesis** **3.** *Moral reputation maintenance concerns moderate the negative association between moral pride and abusive supervision such that this association will be stronger for leaders with higher (vs. lower) moral reputation maintenance concerns.*

**Figure 1:**
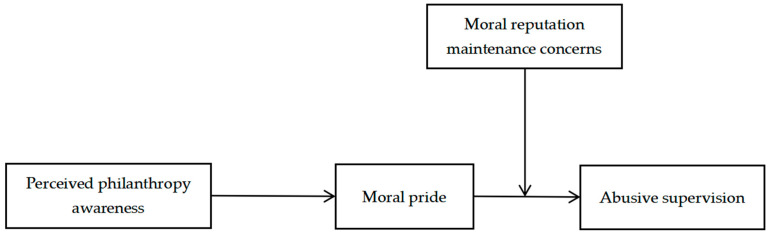
Theoretical model.

Integrating the arguments above, we propose a moderated mediation model (see Figure 1). The indirect effect of philanthropy awareness on abusive supervision via moral pride is hypothesized to be conditional on leaders’ MRMCs. Specifically, the entire preventative pathway—from perceiving the organization’s good deeds to refraining from bad deeds—is hypothesized to be more potent for leaders who care deeply about their moral standing. For these high-MRMC leaders, philanthropy awareness generates pride, which, in turn, powerfully motivates them to act in a reputation-consistent manner by inhibiting abusive behavior. For low-MRMC leaders, this indirect effect will be weaker because the second stage of the mediation (the link from pride to behavior) is attenuated.

**Hypothesis** **4.** *Moral reputation maintenance concerns moderate the indirect effect of perceived philanthropy awareness on abusive supervision via moral pride such that this indirect effect will be stronger when moral reputation maintenance concerns are high.*

## 3. Method

### 3.1. Study Procedure and Participants

A three-wave longitudinal survey was administered on the Prolific online platform. Utilizing a purposive sampling approach, we specifically targeted supervisors (i.e., those who manage one or more subordinates) for data collection, consistent with previous research (e.g., Keng-Highberger et al., 2024; Hu et al., 2024). In our survey, there were no restrictions placed on the participants’ occupations, industries, and countries, aiming to capture a broad spectrum of perceived corporate philanthropy (Luo et al., 2020; Serrano Archimi et al., 2018; He et al., 2025). The study sample demonstrated geographical diversity, drawing participants from 36 distinct countries distributed across six continents (see Table 1). We also included attention check questions (e.g., “Please choose strongly disagree.”) to ensure that the participants were attentive and eligible for the study. The participants were paid a total of GBP 2.89 for completing all three waves of the survey (GBP 0.94 for the Time 1 survey, GBP 0.95 for the Time 2 survey, and GBP 1.00 for the Time 3 survey).

At Time 1, we asked the participants to rate their perceived philanthropy awareness. We received 600 complete responses, of which 573 passed the attention check questions. At Time 2, which took place 7 days after Time 1, we asked the participants to rate their moral pride, social desirability, and moral reputation maintenance concerns. Among the 573 participants who completed the Time 1 survey, 556 provided complete responses to the Time 2 survey. At Time 3, which took place 7 days after Time 2, we asked the participants to disclose abusive supervision behaviors and demographic information. The data from the three time points were matched using the participants’ Prolific IDs. After excluding missing data and removing responses from duplicate IP addresses (Aust et al., 2013), we obtained 434 usable and matched responses across the three waves, yielding a response rate of 72.33%. All questionnaires used in the three surveys were validated psychometric instruments.

Before commencing the surveys, the participants received an informed consent statement detailing the study’s general domain, the voluntary nature of their participation, and the confidentiality of their responses. Completing the questionnaires was deemed to signify consent.

This research obtained ethical clearance from the Institutional Research Ethics Committee of the authors’ university. All study procedures adhered to the ethical principles specified in the Declaration of Helsinki (World Medical Association, 2013).

The study sample consisted of 434 participants. Of these, 219 were men and 215 were women; 86.2% held a bachelor’s degree or above (see Table 2). Their average age was 36.57 years (SD = 11.95), and the average organizational tenure was 7.27 years (SD = 6.11).

### 3.2. Measurements

To assess the core constructs in this study, we used validated instruments, each demonstrating strong internal consistency. Unless otherwise indicated, all the variables were measured on a 5-point Likert-type scale (1 =strongly disagree, 5 = strongly agree). See Appendix A for the complete set of questionnaires.

Perceived philanthropy awareness: We measured perceived philanthropy awareness using a three-item scale adapted from Walker and Kent (2013). In this scale, participants were asked to indicate the extent to which they agree with statements such as “I know of the good things my company does for the community” and “I am aware of the philanthropy of my company”. The internal consistency of this scale was high for our sample (α = 0.88).

Moral pride: We measured moral pride by adapting Boezeman and Ellemers’s (2007) 3-item scale. Sample items are “I feel good when people describe me as a moral person” and “I am proud of being moral”. In the current sample, internal consistency was satisfactory (α = 0.87).

Moral reputation maintenance concerns (MRMCs): We measured moral reputation maintenance concerns using Baer et al.’s (2015) four-item scale. A sample item is “I worry about protecting my reputation” The scale proved to be highly reliable (α = 0.85).

Abusive supervision: We followed previous research’s approach to measuring leaders’ abusive supervision intention (e.g., M. Huang et al., 2023), using Mitchell and Ambrose’s (2007) five-item abusive supervision scale, which was adapted from the abusive supervision scale of Tepper (2000). Participants were asked to respond to sample items such as “I ridicule my subordinates” and “I tell my subordinate his/her thoughts or feelings are stupid”. The internal consistency of this scale was good for our sample (α = 0.88).

Control variables: We controlled for leader demographic variables, including gender, age, and years of formal education, as previous meta-analyses (e.g., Mackey et al., 2017) suggest that leader demographic variables may be related to abusive supervision. It is acknowledged that the key self-report measures, particularly those assessing negative, socially charged behavior are susceptible to social desirability bias (Grimm, 2010). To address this potential threat to validity, we included a validated social desirability scale (α = 0.81) as a control variable in our main analyses—Strahan and Gerbasi’s ten-item scale (M-C 2(10)) (e.g., “I am always courteous, even to people who are disagreeable”). Participants were requested to respond to each item on a 7-point scale ranging from “1= Absolutely false” to “7 = Absolutely true”.

## 4. Results

### 4.1. Measurement Model Validation

We conducted confirmatory factor analysis (CFA) using the lavaan package (Rosseel, 2012) in R to examine measurement validity. The CFA results suggested that the hypothesized four-factor model fit the data well (χ^2^(84) = 229.11, CFI = 0.96, TLI = 0.95, RMSEA = 0.06, SRMR = 0.05). The model also demonstrated good potential for cross-validation (ECVI = 0.69). As presented in Table 3, we compared this hypothesized model with plausible alternative models. The results revealed that the four-factor model provided a significantly better fit to the data than the three-factor model (Δχ^2^(3) = 667.16, *p* < 0.001), the two-factor model (Δχ^2^(5) = 1374.52, *p* < 0.001), and the one-factor model (Δχ^2^(6) = 2549.34, *p* < 0.001), thus confirming the hypothesized factor structure.

Next, we assessed the reliability and validity of the four-factor model, and the results are summarized in Table 4. All constructs displayed excellent internal consistency, with the Composite Reliability (CR) values ranging from 0.85 to 0.89, which are well above the 0.70 threshold. Convergent validity was also supported, as the Average Variance Extracted (AVE) for each factor (ranging from 0.59 to 0.72) exceeded the recommended 0.50 level. We confirmed discriminant validity using the Fornell–Larcker criterion; as shown in Table 4, the square root of each factor’s AVE (bolded values on the diagonal) was greater than its correlation with any other factors.

We assessed the potential for common method variance (CMV) using a two-pronged statistical approach. First, as a preliminary test, we conducted Harman’s single-factor test (Podsakoff & Organ, 1986). Our CFA results (see Table 3) confirmed that the hypothesized four-factor model (χ^2^(84) = 229.11, CFI = 0.960) provided a significantly better fit to the data than the one-factor model where all items loaded onto a single construct (χ^2^(90) = 2778.45, CFI = 0.253). The significant difference (Δχ^2^(6) = 2549.34, *p* < 0.001) provided an initial indication that CMV was not a pervasive issue. Second, CMV was assessed by controlling for the effects of an unmeasured latent method factor, a procedure recommended by Podsakoff et al. (2003). Following this procedure, the inclusion of the latent method factor resulted in a model with only a slightly improved fit to the data (Δχ^2^(15) = 115.89, *p* < 0.001). However, two key indicators suggested this statistically significant improvement did not reflect problematic method variance. First, the latent method factor accounted for only a very small portion (4.46%) of the average item variance. Second, consistent with the CMV diagnostics used in prior research (Jiang et al., 2018), the difference in the Comparative Fit Index between the models (ΔCFI = 0.028) was well below the commonly accepted threshold of 0.050, further indicating that method effects were negligible. In sum, both statistical procedures indicated that common method bias was not a problem in this study.

Finally, we performed a basic measurement invariance check across the high (n = 206) and low (n = 228) MRMC groups (split via median). We compared a configural invariance model (χ^2^(168) = 308.69) with a metric invariance model (constraining factor loadings to be equal across groups; χ^2^(179) = 325.04). The chi-square difference test comparing these nested models was non-significant (Δχ^2^(11) = 16.35, *p* = 0.13), supporting metric invariance and confirming the stability of our measurement model across groups.

### 4.2. Descriptive Statistics and Simple Correlations

Table 5 presents the descriptive statistics and correlations for the variables used in our study. The results showed that perceived philanthropy awareness was positively related to moral pride (*r* = 0.31, *p* < 0.001), which was negatively related to abusive supervision (*r* = −0.21, *p* < 0.001). These findings provide initial support for Hypotheses 1 and 2.

### 4.3. Mediation Effect Analysis

We applied the PROCESS v4.2 macro by Hayes (2017) to conduct the proposed mediation, moderation, and moderated mediation analyses. The results of the regression analyses are shown in Table 6. All reported 95% confidence intervals (CIs) were calculated using a bias-corrected bootstrapping method with 5000 bootstrap samples.

To investigate whether perceived philanthropy awareness is positively related to moral pride and whether the effect of perceived philanthropy awareness on abusive supervision is mediated by moral pride, Model 4 in the SPSS 26 PROCESS v4.2 macro was employed. The results of Model 1 (Table 6) showed that perceived philanthropy awareness was positively related to moral pride (*b* = 0.15, *p* < 0.001, 95% CI = [0.089, 0.216]). Thus, Hypothesis 1 was supported. Hypothesis 2 proposed that moral pride would mediate the relationship between perceived philanthropy awareness and abusive supervision. As can be seen from the results of Model 3 in Table 6, moral pride was negatively related to abusive supervision (*b* = −0.16, *p* < 0.001, 95% CI = [−0.241, −0.070]). The indirect effect was significant (effect = −0.024, 95% CI = [−0.044, −0.009]) and the completely standardized indirect effect was also significant (effect = −0.041, 95% CI = [−0.075, −0.015]), supporting Hypothesis 2.

### 4.4. Moderation Effect Analysis

**Figure 2:**
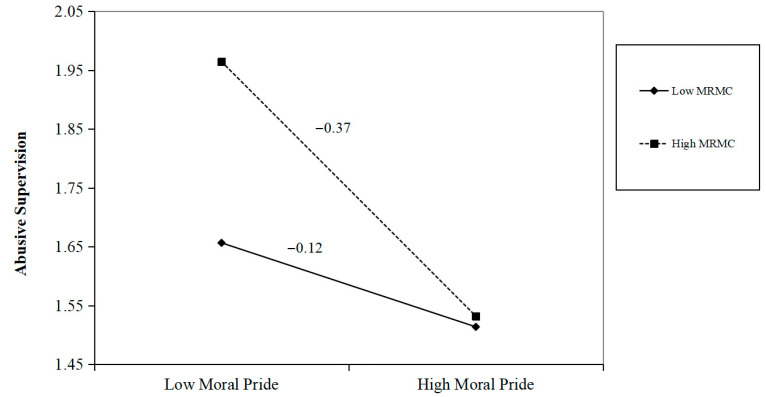
Simple slope analysis results. MRMCs—moral reputation maintenance concerns.

To investigate the moderation effect (Hypothesis 3), Model 14 in the SPSS 26 PROCESS v4.2 macro was employed. Similarly to the above, we calculated the 95% CIs using the bias-corrected bootstrapping method with 5000 bootstrap samples. Hypothesis 3 predicted that moral reputation maintenance concerns would moderate the relationship between moral pride and abusive supervision. From the results of Model 4 in Table 6, we can see that the interaction between moral pride and MRMCs was significantly related to abusive supervision (*b* = −0.12, *p* < 0.001, 95% CI = [−0.19, −0.05]). We conducted a simple slope analysis (Aiken & West, 1991) to help interpret these results. The simple slope analysis showed that the relationship between moral pride and abusive supervision was stronger when leader MRMCs were high (+1 SD) (*b* = −0.37, *p* < 0.001, 95% CI = [−0.51, −0.24]) compared with when they were low (−1 SD) (*b* = −0.12, *p* < 0.05, 95% CI = [−0.22, −0.02]; see Figure 2). Additionally, the difference between the two slopes was significant (*Δb* = −0.25, *p* < 0.001), supporting Hypothesis 3.

### 4.5. Moderated Mediation Effect Analysis

Finally, Model 14 in the SPSS 26 PROCESS v4.2 macro was used to test the moderation mediation effect. Hypothesis 4 proposed that moral reputation maintenance concerns would moderate the indirect effect of perceived philanthropy awareness on abusive supervision through moral pride. The results indicated that the indirect effect of perceived philanthropy awareness on abusive supervision was significant when MRMCs were high (indirect effect = −0.06, SE = 0.02, 95% CI = [−0.095, −0.025]), and it was also significant when MRMCs were low (indirect effect = −0.02, SE = 0.01, 95% CI = [−0.035, −0.005]). These two indirect effects were significantly different from each other, as indicated by an index of moderated mediation of −0.02 based on Hayes (2015) (95% CI = [−0.034, −0.007]). Taken together, these results provided support for Hypothesis 4.

## 5. Discussion

This study investigated a positive, leader-centric pathway to reducing abusive supervision. Drawing on Affective Events Theory, we examined how leaders’ awareness of their organization’s philanthropy could trigger moral pride, which in turn would inhibit abusive behaviors, particularly for those with strong concerns about their moral reputation.

Our findings from a three-wave survey study involving 434 leaders provide robust support for our theoretical model. The zero-order correlations between the study variables were generally consistent in direction and significance with the standardized regression coefficients observed in the subsequent multivariate analyses, further strengthening the initial empirical support for our theoretical model. As hypothesized, perceived philanthropy awareness was positively related to leaders’ moral pride, which, in turn, mediated the negative relationship between perceived philanthropy awareness and abusive supervision. Furthermore, this indirect, preventative effect was stronger for leaders with high moral reputation maintenance concerns.

### 5.1. Theoretical Implications

Our research offers several important theoretical contributions. First, we contribute to the literature on abusive supervision by shifting the focus from negative, resource-depleting antecedents to positive, resource-generating ones. By identifying leaders’ awareness of corporate good deeds as a deterrent to individual bad deeds, we introduce a novel preventative mechanism rooted in the positive aspects of the organizational context. This actor-centric approach (Qin et al., 2018; Ju et al., 2019) answers calls to understand the upstream influence of organizational context on leader behavior and complements the dominant self-regulation models of abusive supervision (e.g., Barnes et al., 2015) by highlighting a process of moral fortification rather than focusing solely on resource depletion. By locating our study within a multi-level framework, we demonstrate how a meso-level organizational practice (philanthropy) influences micro-level leader outcomes (Cha & Rajadhyaksha, 2021).

Second, we extend the application of Affective Events Theory (Weiss & Cropanzano, 1996) to the domain of leadership ethics. We demonstrate that a macro-level organizational practice (philanthropy) can serve as a potent affective event for leaders, influencing their individual moral emotions and, consequently, their interpersonal behaviors. This provides a clear micro-foundational link between corporate social responsibility and leadership quality, showing how “doing good” at the organizational level can inspire leaders to “do no harm” at the dyadic level.

Third, our study contributes to the literature on moral emotions by elucidating the self-regulatory function of moral pride in the leadership context. While pride is often studied for its effects on performance or motivation (Williams & DeSteno, 2008), we demonstrate its role in inhibiting destructive and unethical behaviors. Our findings suggest that moral pride is not merely a pleasant feeling but a motivational state that, especially when coupled with reputational concerns, encourages behavioral consistency with one’s moral self-concept. The moderation effect of MRMCs highlights the social and self-presentational components of moral emotions, illustrating that the behavioral consequences of such emotions are amplified when one’s moral identity is on the line.

### 5.2. Practical Implications

Our findings suggest a potentially low-cost intervention framework for organizations seeking to cultivate more ethical and effective leadership while reducing abusive supervision. This framework primarily leverages existing organizational practices and focuses on reinforcing positive leader psychology. First, organizations should enhance leaders’ awareness of corporate good deeds. This involves moving beyond viewing philanthropy solely as an external relations tool and instead systematically and routinely communicating philanthropic initiatives and their positive impacts internally through various channels, such as newsletters, meetings, and intranet updates. Furthermore, organizations can deepen this effect by facilitating direct or indirect contact between leaders (and potentially employees) and the beneficiaries of the company’s philanthropic efforts. Research suggests that such contact can significantly amplify positive affective responses like moral pride and strengthen positive attitudes toward the organization (Block et al., 2017).

Second, communications about philanthropic initiatives should explicitly link these activities to the organization’s core ethical values and the desired moral identity of its leaders, which will help to internalize the meanings of these “good deeds”. Concurrently, recognizing that leaders differ in their moral reputation maintenance concerns (MRMCs), organizations can tailor their approach. For leaders with high MRMCs, subtly highlighting how ethical conduct, including non-abusiveness, is vital for maintaining a respected moral reputation can be motivating. Public recognition and rewards for ethical leadership could further strengthen this connection. Leadership development programs might also incorporate modules aimed at cultivating positive moral emotions, such as moral pride.

Third, implementing such initiatives requires follow-up. Organizations should monitor the interpersonal climate post-intervention, perhaps through anonymous upward feedback or pulse surveys, to assess whether these interventions are translating into tangible improvements in leader behavior and subordinate well-being. This allows for data-driven refinement of communication and development strategies.

It is crucial to proactively address the potential risk of moral licensing (Merritt et al., 2010; Sachdeva et al., 2009). Paradoxically, heightened awareness of the organization’s “goodness” through philanthropic initiatives might lead some leaders to feel psychologically entitled to engage in minor transgressions, such as abusive supervision, believing that the organization’s positive actions create a moral buffer. To safeguard against this, organizations must align their narratives. Communications celebrating philanthropy should be explicitly paired with reinforcement of clear organizational norms and policies condemning abusive supervision. The message of these communications must consistently conveys that external good deeds do not excuse poor internal conduct. Crucially, this message must be backed by robust accountability mechanisms within performance management and reward systems. By managing awareness, reinforcing moral identity, monitoring outcomes, and actively safeguarding against moral licensing, organizations can more effectively translate their external pro-social investments into a healthier and more ethical internal leadership environment.

### 5.3. Limitations and Future Directions

Despite its strengths, including a three-wave survey design, this study has limitations that offer avenues for future research. First, our data were collected via Prolific, an online participant platform. We acknowledge that the use of a non-probability convenience sample drawn from Prolific limits the generalizability of our findings. While recent empirical research suggests Prolific can provide high-quality data (Palan & Schitter, 2018; Peer et al., 2017), our sample does not constitute a representative sample of all leaders. Furthermore, our sample includes leaders from mixed sectors, making it challenging to generalize our results to any single industry without further investigation. Although Prolific allowed us to efficiently target individuals holding supervisory roles, future research should aim to replicate this study using organizationally embedded samples to enhance contextual validity and conduct multi-sector replications, potentially with probability sampling methods where feasible, to better understand potential variations across different organizational contexts.

Second, our reliance on self-reported measures may raise concerns about common method bias and social desirability (Podsakoff et al., 2003). To mitigate this, we controlled for social desirability bias using a validated scale (Strahan & Gerbasi, 1972) and implemented a three-wave design to temporally separate the measurement of the predictors, mediator, and outcome. While these steps help to reduce bias, we acknowledge that objective or multi-source data—such as subordinate ratings of abusive supervision or archival CSR metrics—could further strengthen the research.

Third, while our three-wave design provides temporal precedence for the proposed causal direction, we must acknowledge the possibility of reverse or alternative causality. To more rigorously establish causality, future studies could employ cross-lagged panel designs examining the hypothesized relationships over multiple, extended time points. Alternatively, experimental designs could provide stronger causal evidence. Furthermore, while our proposed intervention framework offers practical steps, its successful implementation is likely contingent upon various micro-level factors, particularly the characteristics of the leaders themselves. Leaders with significant personality difficulties may hinder organizational progress toward adopting pro-social and ethical initiatives. For example, leaders with high levels of narcissism might actively resist efforts centered on collective good, moral introspection, or subordinate well-being, prioritizing personal gain, status, and control over institutional objectives and ethical considerations (O’Boyle et al., 2012; Wisse & Sleebos, 2016). Such personalities may be less receptive to the positive reinforcement derived from philanthropy awareness or moral identity exercises, viewing them cynically or instrumentally. Therefore, organizations can shift performance evaluations to heavily weigh observable interpersonal behaviors (e.g., using a Behaviorally Anchored Rating Scale (BARS)) and subordinate feedback (e.g., 360-degree reviews), rather than just task outcomes. Future research could explore how specific leader personality traits moderate the effectiveness of such interventions, and intervention providers should consider leader selection, targeted development, and potentially stronger accountability systems for leaders whose individual characteristics may make them resistant to softer, awareness-based approaches.

Finally, this study focused on leaders’ perceived awareness of corporate philanthropy but did not examine other contextual factors such as internal CSR practices, ethical climate, or organizational justice, which may interact with or amplify the observed effects (Newman et al., 2017). Future research could adopt a multilevel or configurational lens to explore how internal and external CSR initiatives jointly influence ethical leadership across diverse organizational and cultural settings.

## 6. Conclusions

In conclusion, our study provides compelling evidence that an organization’s good deeds can have a ripple effect inwardly, shaping the emotional and behavioral tendencies of its leaders. By demonstrating that leaders’ awareness of corporate philanthropy can foster moral pride and thereby reduce abusive supervision, we illuminate a positive pathway to better leadership. The finding that this effect is stronger for leaders who care about their moral reputation underscores the powerful interplay between feeling good, doing good, and the fundamental human desire to be seen as a good person.

## Figures and Tables

**Table 1:** Geographical distribution of the sample (N = 434).

Continent	N (Frequency)	Percentage (%)
Africa	208	47.93
Asia	8	1.84
Europe	147	33.87
North America	61	14.06
Oceania	5	1.15
South America	5	1.15
Total	434	100%

**Table 2:** Demographic characteristics of the sample (N = 434).

Characteristic	Category	N (Frequency)	Percentage (%)
Gender	Male	219	50.5
	Female	215	49.5
Age (Years)	19–30	166	38.2
	31–45	173	39.9
	46–60	74	17.1
	≥61	21	4.8
Education	High school diploma and below	42	9.7
	Associate’s degree	18	4.1
	Bachelor’s degree	211	48.6
	Master’s degree	129	29.7
	Doctoral degree	34	7.8
Tenure (Years)	0–3	134	30.9
	4–10	207	47.7
	≥11	93	21.4

**Table 3:** Confirmatory factor analysis results.

Model	χ^2^	d*f*	*p*	CFI	TLI	RMSEA	SRMR	ECVI
Hypothesized four-factor model	229.11	84.00	—	0.96	0.95	0.06	0.05	0.69
Three-factor model (PPA + MP, AS, MRMC)	896.27	87.00	<0.001	0.78	0.73	0.15	0.12	2.22
Two-factor model (PPA + MP + MRMC, AS)	1603.63	89.00	<0.001	0.58	0.50	0.20	0.18	3.84
One-factor model	2778.45	90.00	<0.001	0.25	0.13	0.26	0.25	6.54

PPA—perceived philanthropy awareness, MP—moral pride, MRMC—moral reputation maintenance concerns, AS—abusive supervision; “+” means that the variables were combined.

**Table 4:** Descriptive statistics, reliability, validity, and correlations among latent variables.

Variable	CR	AVE	1	2	3	4
1. PPA	0.89	0.72	(0.85)			
2. MP	0.88	0.71	0.35 ***	(0.84)		
3. MRMC	0.85	0.59	0.15 **	0.31 ***	(0.77)	
4. AS	0.88	0.60	−0.07	−0.21	0.10	(0.77)

PPA—perceived philanthropy awareness, MP—moral pride, MRMC—moral reputation maintenance concerns, AS—abusive supervision. ** *p* < 0.01, *** *p* < 0.001.

**Table 5:** Descriptive statistics and correlations.

	Mean	SD	1	2	3	4	5	6	7	8
1. Gender	0.50	0.50								
2. Age	36.57	11.95	−0.01							
3. Education	3.22	1.00	0.12 *	0.01						
4. Organizational tenure	7.27	6.11	−0.03	0.62 **	0.00					
5. Social desirability	4.94	1.03	0.04	0.07	0.12 *	0.05				
6. Perceived philanthropy awareness	3.90	0.90	0.19 ***	−0.10 *	0.10 *	−0.03	0.33 ***			
7. Moral pride	4.42	0.59	0.07	−0.06	0.07	−0.08	0.27 ***	0.31 ***		
8. Moral reputation maintenance concerns	3.41	1.01	0.12 **	−0.09 *	0.02	−0.09	−0.08	0.14 **	0.28 ***	
9. Abusive supervision	1.30	0.52	0.07	−0.09	−0.04	−0.07	−0.19 ***	−0.09	−0.21 ***	0.11 *

N = 434. Gender: 0—male; 1—female. Education: 1—high school diploma and below; 2—associate’s degree; 3—bachelor’s degree; 4—master’s degree; 5—doctoral degree. Tenure was measured in years. * *p* < 0.05, ** *p* < 0.01, *** *p* < 0.001.

**Table 6:** Regression analysis results.

	Moral Pride			Abusive Supervision								
	Model 1			Model 2			Model 3			Model 4		
	*b*	*S*.*E*.	*t*	*b*	*S.E.*	*t*	*b*	*S.E.*	*t*	*b*	*S.E.*	*t*
Gender	0.02	0.05	0.39	0.10	0.05	1.90	0.10	0.05	1.99 *	0.08	0.05	1.69
Age	−0.00	0.00	−0.07	−0.00	0.00	−1.20	−0.00	0.00	−1.22	−0.00	0.00	−1.11
Education	0.01	0.03	0.52	−0.01	0.03	−0.42	−0.01	0.02	−0.33	−0.00	0.02	−0.14
Tenure	−0.01	0.01	−1.29	−0.00	0.01	−0.29	−0.00	0.01	−0.52	−0.00	0.00	−0.43
Social desirability	0.11	0.03	4.04 ***	−0.09	0.03	−3.35 **	−0.07	0.03	−2.64 **	−0.05	0.03	−1.94
Perceived philanthropy awareness	0.15	0.03	4.73 ***	−0.03	0.03	−1.04	−0.01	0.03	−0.23	−0.01	0.03	−0.38
Moral pride							−0.16	0.04	−3.57 ***	−0.24	0.05	−5.14 ***
Moral reputation maintenance concerns										0.08	0.03	3.27 **
Moral pride × MRMCs										−0.12	0.04	−3.32 ***
R-SQUARE	0.13			0.05			0.08			0.12		
ΔR^2^							0.03			0.07		

N = 434. 0—male, 1—female. MRMCs—moral reputation maintenance concerns. Unstandardized regression coefficients are reported. * *p* < 0.05, ** *p* < 0.01, *** *p* < 0.001. Note: Robustness checks were conducted to ensure model stability. Specifically, we re-ran all analyses (1) by introducing geographical controls (continent dummy variables) to the baseline model, and (2) by excluding social desirability while retaining the geographical controls. In both instances, all four hypothesized relationships remained statistically significant and consistent with the reported findings, confirming the robustness of the theoretical model.

## Data Availability

The data presented in this study are available from the corresponding author upon request.

## Appendix A

The following scales were used in our study:

Perceived philanthropy awareness (Walker & Kent, 2013): I know of the good things my company does for the community. It pleases me to know that my company is a charitable organization. I am aware of the philanthropy of my company.

Moral pride (Boezeman & Ellemers, 2007): I feel good when people describe me as a moral person. I am proud of being moral. I am proud to be a person with a sense of morality.

Moral reputation maintenance concerns (Baer et al., 2015): I’m concerned about maintaining my image. I worry about protecting my reputation. I feel the need to preserve the opinion others have of me. I’m pre-occupied with keeping others’ views of my character intact.

Abusive supervision (Mitchell & Ambrose, 2007): I ridicule my subordinates. I tell my subordinate his/her thoughts or feelings are stupid. I put one of my subordinates down in front of others. I make negative comments about my subordinates to others. I tell my subordinate he/she is incompetent.

Social desirability (Strahan & Gerbasi, 1972) I never hesitate to go out of my way to help someone in trouble. I have never intensely disliked anyone. When I don’t know something I don’t at all mind admitting it. I am always courteous, even to people who are disagreeable. I would never think of letting someone else be punished for my wrong doings. I sometimes feel resentful when I don’t get my way. (R) There have been times when I felt like rebelling against people in authority even though I knew they were right. (R) I can remember “playing sick” to get out of something. (R) There have been times when I was quite jealous of the good fortune of others. (R) I am sometimes irritated by people who ask favors of me. (R)
